# Usage and Feasibility of Web Applications in Speech‐Language Therapy: Insights from the Communication Bridge 2 Trial for Primary Progressive Aphasia

**DOI:** 10.1002/alz70863_110899

**Published:** 2025-12-23

**Authors:** Ollie Fegter, Angela C. Roberts, Matthew Bona, Alfred Rademaker, Emily J Rogalski

**Affiliations:** ^1^ Northwestern University Feinberg School of Medicine, Chicago, IL USA; ^2^ University of Chicago, Chicago, IL USA; ^3^ Western University, London, ON Canada; ^4^ Northwestern University, Chicago, IL USA; ^5^ Healthy Aging & Alzheimer's Research Care (HAARC) Center, Healthy Aging & Alzheimer's Research Care (HAARC) Center, The University of Chicago, Chicago, IL USA

## Abstract

**Background:**

Primary Progressive Aphasia (PPA) is a clinical dementia syndrome characterized by progressive language decline. Access to care for individuals living with PPA is limited by a shortage of evidence‐based interventions and qualified clinicians. While technology‐supported approaches show promise in improving access to care, there has been no systematic exploration of the factors affecting web application use in this population. This study evaluated the *usage* and *feasibility* of an app‐based intervention through quantitative application engagement data and qualitative insights from semi‐structured post‐study interviews.

**Method:**

Participants enrolled in Communication Bridge‐2 (NCT03371706, *n* = 95), a 12‐month NIH stage 2 randomized controlled trial of speech‐language therapy for PPA, were encouraged to complete app‐based home practice exercises five days per week for 30 minutes per day. *Usage* was measured by weekly logins and completed home practice exercises. *Feasibility* was assessed through semi‐structured post‐study interviews (PSI; *n* = 79). Multi‐methods analysis incorporated descriptive statistics and thematic coding of qualitative data.

**Result:**

On average, participants logged into the app 5.88 times per week (SD=1.29, range 1.29‐18.90) to access exercises and educational materials across the ∼12‐month duration of the intervention. On average, users completed 13.7 home practice exercises (SD=5.95) across 3.99 days per week (SD=1.19). PSI dyadic reports indicated few technical challenges with the application (*n* = 76, 85%) with the most common issues including software updates (*n* = 12) or connectivity problems (*n* = 10). Most (*n* = 80, 90%) found the computer‐based format helpful, and many (*n* = 26, 29%) described the app as user‐friendly and intuitive after initial training. Participants highlighted the app's role in fostering confidence and motivation for home communication practice.

**Conclusion:**

This study demonstrates high *usage* and *feasibility* of web applications to support communication and language intervention in PPA. Importantly, all participants logged into the app and completed home practice exercises at least weekly. Most participants experienced minimal technical challenges and found the web‐based format easy to use following a brief technology orientation. These findings challenge assumptions about technology use in older adults with cognitive‐communication impairments and provide support for web applications as viable tools for supporting evidence‐based speech‐language home practice exercises and educational videos to individuals with PPA and their communication partners.

